# Day-to-day variability in activity levels detects transitions to depressive symptoms in bipolar disorder earlier than changes in sleep and mood

**DOI:** 10.1186/s40345-025-00379-6

**Published:** 2025-04-02

**Authors:** Abigail Ortiz, Ramzi Halabi, Martin Alda, Alexandra DeShaw, Muhammad I. Husain, Abraham Nunes, Claire O’Donovan, Rachel Patterson, Benoit H. Mulsant, Arend Hintze

**Affiliations:** 1 Department of Psychiatry, Temerty Faculty of Medicine, University of Toronto, Toronto, ON Canada; 2 Campbell Family Research Institute, Centre for Addiction and Mental Health (CAMH), Toronto, ON Canada; 3 Department of Psychiatry, Dalhousie University, Halifax, NS Canada; 4 National Institute of Mental Health, Klecany, Czech Republic; 5 Department of MicroData Analytics, Dalarna University, Dalarna, Sweden

**Keywords:** Bipolar disorder, Wearable technology, Densely-sampled, Mood variability, Activity, Sleep, Onset

## Abstract

Anticipating clinical transitions in bipolar disorder (BD) is essential for the development of clinically actionable predictions. Our aim was to determine what is the earliest indicator of the onset of depressive symptoms in BD. We hypothesized that changes in activity would be the earliest indicator of future depressive symptoms. The study was a prospective, observational, contactless study. Participants were 127 outpatients with a primary diagnosis of BD, followed up for 12.6 (5.7) [(mean (SD)] months. They wore a smart ring continuously, which monitored their daily activity and sleep parameters. Participants were also asked to complete weekly self-ratings using the Patient Health Questionnaire (PHQ-9) and Altman Self-Rating Mania Scale (ASRS) scales. Primary outcome measures were depressive symptom onset detection metrics (i.e., accuracy, sensitivity, and specificity); and detection delay (in days), compared between self-rating scales and wearable data. Depressive symptoms were labeled as two or more consecutive weeks of total PHQ-9 > 10, and data-driven symptom onsets were detected using time-frequency spectral derivative spike detection (TF-SD^2^). Our results showed that day-to-day variability in the number of steps anticipated the onset of depressive symptoms 7.0 (9.0) (median (IQR)) days before they occurred, significantly earlier than the early prediction window provided by deep sleep duration (median (IQR), 4.0 (5.0) days; *p* <.05). Taken together, our results demonstrate that changes in activity were the earliest indicator of depressive symptoms in participants with BD. Transition to dynamic representations of behavioral phenomena in psychiatry may facilitate episode forecasting and individualized preventive interventions.

## Introduction

Conceptualizing changes in symptoms and behaviors using dynamic approaches is not new (Hargreaves and Blacker 1967; Odgers et al. 2009), yet symptoms continue to be measured as static indicators. This limitation explains in part the modest accuracy of prediction models for suicide (Franklin et al. 2017), recurrence of depressive episodes (Moriarty et al. 2021) or transition to full psychotic syndromes (Fusar-Poli et al. 2013). Several theoretical models, including dynamic systems theory and network theory, among others (Nelson et al. 2017) have been suggested as a framework to characterize the processes of symptom variation in self-regulated dynamical systems. In schizophrenia, for instance, positive and negative symptoms show different temporal patterns of exacerbation (Arndt et al. 1995). However, it has been challenging to determine the dynamics of symptom variation in mood disorders, partially because we do not understand how symptoms evolve before transitioning into a full episode. In other words, we do not yet know how the earliest expressions of mood disorders manifest, e.g., whether it is in the form of mood changes that cascade into changes in activity or vice versa.

Over the past ten years, studies using ecological momentary assessments (EMA) have reported time-lagged associations between activity and positive affect (Giurgiu et al. 2019; Wichers et al. 2012); negative affect and paranoia (Kramer et al. 2014); or sleep and mood (de Wild-Hartmann et al. 2013) in the general population. Time-lagged associations between activity and mood have also been documented in patients with other general medical conditions, including pelvic pain (Naliboff et al. 2021), stroke (Bui et al. 2022; Forster et al. 2022), and fatigue associated with hemodialysis (Brys et al. 2021). In bipolar disorder (BD), studies have described time-lagged associations between sleep and mood (Patapoff et al. 2022), impulsivity and anxiety (Titone et al. 2022), and activity and mood (Ortiz et al. 2018). Two studies combining actigraphy and EMA for three weeks found a unidirectional association between motor activity and mood (Merikangas et al. 2019; Walsh et al. 2022), suggesting that interventions targeting activity might be even more relevant than previously thought to treat mood disorders.

Other authors have also investigated the potential of smartphone data to objectively measure behavioral patterns in BD (Faurholt-Jepsen et al. 2014, 2015, 2019; Hidalgo-Mazzei et al. 2018); as well as the role of EMA in capturing real-time mood fluctuations (Ebner-Priemer and Trull 2009). Several groups have further advanced the concept of digital data collection, advocating for a comprehensive and clinically-relevant models to enhance the replicability of findings using densely-sampled data (Ebner-Priemer et al. 2020; Wadle and Ebner-Priemer 2023).

More recently, studies have started to focus on anticipating transitions to full syndromic illness by analyzing the temporal structure of longitudinal symptoms: in a recent study in 36 patients diagnosed with schizophrenia, where self-reported sleep duration and clinical symptoms were assessed once a day for over a year, reduced sleep duration anticipated exacerbation of psychotic symptoms by 1–12 days (Meyer et al. 2022). However, studies in small samples of patients with major depressive disorder did not observe patterns suggestive of a future transition to a depressive episode (Bos and De Jonge 2014). Similarly, in two recent studies including up to 15 participants with BD followed for 4–6 months using EMA (Bos et al. 2022) or actigraphy (Kunkels et al. 2021), there was poor agreement between metrics prior to detecting a transition. Several methodological concerns may explain this lack of success when predicting transitions in BD, including clinical heterogeneity, the inability to detect more complex types of transitions, and the role of noise (Dablander and Bury 2022). None of the studies in BD analyzed the temporal structure of longitudinal symptoms separately, even though a recent meta-analysis identified that activation and mood represent distinct dimensions in BD (Scott et al. 2017). Moreover, it has been shown that both low and high energy levels are predictors of suicidal behavior (Depp et al. 2017) and that mood regulation is a short-term process (Ortiz et al. 2021), for which predictions four weeks in advance are likely to be inaccurate (Dablander and Bury 2022; Kendler et al. 2011). Other concerns include incomplete preprocessing of time-series data, analyzing only one type of data (e.g., subjective or objective), small sample sizes, and short follow-up periods.

Thus, while detecting or anticipating transitions in Psychiatry is an important component of developing sound predictive systems, several clinical and methodological questions need to be addressed prior to incorporating electronic systems that alert patients of potential warning signs, as these may be inaccurate, difficult to interpret, and cause unnecessary stress (Depp et al. 2016). In this context, we conducted a study to detect the onset of depressive symptoms in BD participants followed for over a year, using densely-sampled data. We analyzed whether changes in activity, sleep, or mood were the earliest indicator of the transition to a mood episode, and which of these changes could be detected first. Based on the literature reviewed above, we hypothesized that changes in activity would be the earliest depressive symptom.

## Methods

### Participants

This prospective observational contactless study recruited 127 adult participants diagnosed with BD type I or II between December 12, 2020, and February 1, 2023, at two academic psychiatric hospitals in Canada: The Adult Psychiatry Division, Centre for Addiction and Mental Health (CAMH), Toronto, Ontario; and the Mood Disorders Program, Queen Elizabeth II Health Sciences Centre, Halifax, Nova Scotia. Fully informed by the Privacy Office at both institutions, the Research Ethics Board (REB) approval (# 059-2019) was obtained at each of the above-mentioned academic centers, in accordance with the Declaration of Helsinki.

## Study design

Primary diagnoses were made according to the Diagnostic and Statistical Manual of Mental Disorders 5 (DSM-5) (2013) confirmed by the Structured Clinical Interview for DSM-5 (SCID-5) (First et al. 2015). After all procedures had been fully explained and written consent was obtained, a comprehensive baseline assessment was completed: participants provided information regarding their sociodemographic characteristics, clinical course, cardiovascular status, chronotype, and pharmacotherapy. Race, ethnicity, sex at birth and gender identity were self-reported. To determine the polarity of each participant (i.e., euthymia, depression, or (hypo)mania), the Young Mania Rating Scale (YMRS) (Young et al. 1978) and the Montgomery-Asberg Depression Rating Scale (MADRS) (Montgomery and Asberg 1979) were administered by a trained rater. Euthymia was defined as YMRS ≤ 10 and MADRS ≤ 10 for at least two months; depressive polarity was defined as MADRS ≥ 10 for at least two weeks, and (hypo)manic polarity was defined as YMRS ≥ 10 for at least one week. We chose our cutoffs to reflect real-world data, which in turn improves the generalizability of our results and applicability in non-research settings. We based our decision of a cutoff ≤ 10 in MADRS to define euthymia in these three seminal papers (Frank et al. 1991; Hawley et al. 2002; Zimmerman et al. 2004). Similarly, we based our decision of a cutoff ≤ 10 in YMRS to define euthymia with the understanding that a YMRS score < 10 defines “symptomatic remission”, as per these influential papers (Tohen et al. 2006; Vieta et al. 2008). Participants received treatment according to standard local practices.

After baseline assessment, participants were asked to wear an Oura ring (Oura Health Oy, Generation 2, Oulu, Finland) continuously for the entire duration of the study(Ortiz et al. 2022). This wearable monitors daily activity (e.g., total steps, minutes of inactivity) and sleep parameters (e.g., total duration, REM duration, deep sleep duration). Several studies have validated Oura ring’s sleep tracking algorithms against polysomnography, in healthy adults (Altini and Kinnunen 2021; de Zambotti et al. 2017; Ong et al. 2024; Robbins et al. 2024; Svensson et al. 2024). In the most recent study (Ong et al. 2024) that compared sleep stage detection between PSG, EEG, and commercial wearables, the Oura ring showed an accuracy of 91% when compared to PSG for sleep stages.

Participants were also asked to complete weekly self-ratings using the Patient Health Questionnaire (PHQ-9)(Kroenke et al. 2001) and Altman Self-Rating Scale (ASRS) (Altman et al. 1997) scales. Data was captured using on-premise REDCap (Research Electronic Data Capture) servers (Harris et al. 2009). This data collection scheme generated densely and irregularly sampled multivariate time series data for each participant. Because our analyses focused on detecting transitions from euthymia to depressive symptoms, we excluded participants with a manic, hypomanic or mixed polarity from our analyses. For those participants who entered the study in a depressive episode, we stabilized them first so they could reach euthymia.

### Data preprocessing and labeling

The onset of depressive symptoms was defined as the start of at least two consecutive weeks with a PHQ-9 score ≥ 10, with several studies reporting high sensitivity and specificity at this cutoff (Levis et al. 2019; Stochl et al. 2022). Activity and sleep variables were normalized to (0, 1) bounds to account for inter-variable and inter-participant range variability. Upon normalization, each participant’s data was labeled in a binary fashion such that data patterns not suggestive of depressive symptoms were assigned a label of ‘0’ and data patterns suggestive of depressive symptoms were assigned a label of ‘1’. To account for the sampling rate difference between daily wearable data and weekly self-rating scales, we followed a period-level label filling approach across all days within every period (i.e., assigning a minimum of 14 consecutive positive labels). This approach mitigates the potential onset detection delay due to the two-week offset of self-reported symptoms required for PHQ-9 reporting. Because subjective data in e-monitoring studies is not missing at random (Halabi et al. 2024), no self-rating imputation was performed to account for missing data. To ensure quality control and prevent spurious data non-stationarities affecting our analyses of the wearable data, we customized and performed variable-specific selective mean imputation. This algorithm fills the missing data instances in each variable (e.g., steps, total sleep duration) for each participant with the mean of that variable according to its associated label.

### Depressive symptoms onset detection

#### Temporal scale pattern separation

To extract non-stationary hidden patterns of activity and sleep from raw wearable data that could be indicative of depressive symptoms, we implemented the Complete Ensemble Empirical Mode Decomposition with Additive Noise (CEEMDAN) technique (Colominas et al. 2014), an optimized version of the empirical mode decomposition (EMD) algorithm. The EMD algorithm recursively performs self-adaptive decomposition and pattern separation of non-stationary univariate time series into a set of underlying spectrally ordered intrinsic mode functions. The main feature that renders EMD suitable for use in detecting patterns in longitudinally and densely sampled wearable data is its lossless decomposition, which guarantees that no clinically relevant patterns are altered or lost.

#### Time-Frequency analysis

**Fig. 1:**
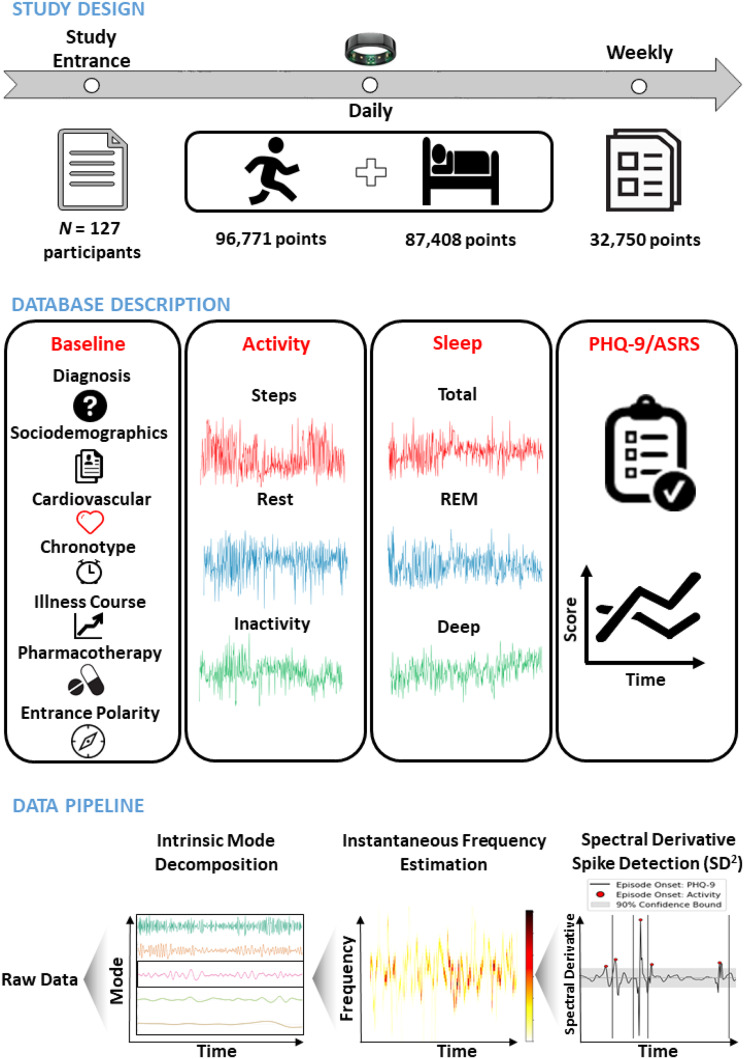
Study design

We computed the Hilbert transform-based instantaneous frequency (IF) (Cizek 1970) of each extracted univariate intrinsic mode function throughout the course of the study, hypothesizing that one or more of these time series carried information about symptom onset and progression dynamics. The time-frequency spectrograms allowed us to observe the temporal progression of activity and sleep data spectra (i.e., the rate of data fluctuation at different frequency levels - e.g., daily, weekly), such that a lower IF indicated lower data variability at a specific time instant, and vice versa. The study design and data pipeline design are presented in Fig. 1.

#### Spectral derivative Spike detection (SD^2^) algorithm

Upon computation of the time-frequency IF signals across variables and participants, we performed rescaling to (-1, 1) bounds using *MinMax* scaling to minimize the effect of inter-variable and inter-participant scale differences. Subsequently, we computed the spectral derivative of the individual IFs to *(a)* minimize the effect of inter- and intra-participant spectral baseline instability, and *(b)* to highlight the onset of significant spectral variability potentially indicative of the onset of depressive symptoms. Finally, to capture the instances at which the IF transitions changed in either direction, we implemented a spectral derivative spike detector (SD^2^) (Ortiz and Halabi 2024), using a tuned peak detection algorithm with a z-score of 1.645 (90% CI) as upper and lower spike prominence thresholds, and a minimum inter-spike interval (ISI) of 14 days. Positive IF spikes were indicative of sharp increases in variability in activity or sleep pattern, while negative IF spikes were indicative of steep decreases in variability. We based the ISI on the PHQ-9-derived minimum duration of a depressive episode, such that any two successive spikes with an ISI of less than 14 days are deemed as a manifestation of the same detected depressive symptoms.

#### Detection performance assessment

To assess the performance of the SD^2^ in detecting depressive symptoms onsets, we used the PHQ-9 as a label (dependent variable), as previously described in the labeling procedure. We assessed whether changes in daily activity or sleep (independent variables) during a two-week window before the onset of depressive symptoms (early detection sweeping, EDS) and one-week window before and after the onset of depressive symptoms (symmetrical bilateral sweeping, SBS) were useful to detect depressive symptoms (defined based on self-reported PHQ-9 ≥ 10, as per above).

Then, we used EDS and SBS to assess our algorithm’s performance across different windows, as follows:

- EDS: A spike in variability was considered an early detection if it occurred up to 2 weeks prior to the onset of depressive symptoms.
- SBS: A spike in variability was considered an early detection if it occurred between one week before and one week after the onset of depressive symptoms.

For these analyses, we focused on 2-week windows based on our previous work (Ortiz et al. 2015, 2016, 2018, 2021) on the nonlinear architecture of mood regulation in BD, which inevitably results in short windows for episode detection or prediction.

For both EDS and SBS, detection performance metrics included sensitivity, specificity, and accuracy across all participants, for each time scale from raw day-to-day to every three weeks data. We used six variables for comparative detection performance: for activity: number of steps, duration of rest (in minutes), and duration of inactivity (in minutes); for sleep: total sleep duration, REM duration, and deep sleep duration (all in minutes). The Oura ring manufacturer defines “steps” as “the total count of steps registered during the day”; “rest” is defined as “number of minutes during the day spent resting, i.e., sleeping or lying down (with the average Metabolic Equivalent of a Task (MET) level of the minute below 1.05)”; and “inactivity” is defined as “number of inactive minutes, i.e., sitting or standing still (with average MET level of the minute between 1.05 and 2.0) during the day”.

### Statistical analysis

We used Kruskal-Wallis and chi-squared tests for continuous and categorical variables, respectively, to assess statistical significance of sociodemographic and clinical variables. We implemented data-driven IF peak detection with a z-score of 1.645 (90% CI) as upper and lower spike prominence thresholds and a minimum inter-spike interval (ISI) of 14 days. For pairwise onset detection delay between each of the activity and sleep variables (independent variables), and the self-rated PHQ-9 score (dependent variable), we used the Mann-Whitney U test, followed by Benjamini-Hochberg p-value correction. To account for within-subject dependencies, we performed separate Mann-Whitney U tests for each participant, comparing the onset detection delays between activity/sleep-based measures and PHQ-9-defined depressive symptoms. To obtain an overall statistical significance across participants, we then applied Fisher’s method to combine the per-subject p-values into a single aggregated p-value. This approach ensured that our statistical analysis respects the hierarchical structure of the data while preserving the advantages of a non-parametric test. All statistical analyses were performed using Python 3.11.

## Results

### Data description

Over 383.5 (175.3) days (mean (SD) duration), 127 participants wore an e-monitoring device. See Table 1 for a detailed description of the participants’ demographic and clinical characteristics. We analyzed 96,771 datapoints for activity (evenly distributed across 3 variables: steps, rest, and inactivity); and 87,408 datapoints for sleep (evenly distributed across 3 variables: total sleep, REM sleep, and deep sleep). See Table 2 for details.

**Table 1:** Study cohort demographic and clinical characteristics

Characteristic	Participants	*P* Value
	Total (*N* = 127)	
Age, mean [SD], y	39.2 [12.6]	0.41
Number of days in the study, mean [SD]	383.5 [175.3]	0.48
Sex assigned at birth, No. (%)		
Female	82 (64.6)	0.45
Male	45 (35.4)	
Gender, No. (%)		
Woman	69 (54.3)	0.45
Preferred not to disclose	13 (10.2)	
Education, No. (%)		
Completed high school or less	24 (18.9)	0.44
Completed 4-year university degree	39 (30.7)	
Completed post-secondary education	64 (50.4)	
Marital status, No. (%)		
Single	65 (51.2)	0.45
Married	42 (33.1)	
Divorced	20 (15.7)	
Socioeconomic status, No. (%)		
Work full-time	64 (50.4)	0.43
Work part-time	13 (10.2)	
Unemployed	15 (11.8)	
On disability or social assistance	18 (14.2)	
Student	9 (7.1)	
Retired	5 (3.9)	
Other	3 (2.4)	
Primary Diagnosis, No. (%)		
Bipolar Disorder I	83 (65.4)	0.46
Bipolar Disorder II	44 (34.6)	
Predominant polarity (lifetime), No. (%)		
Depressive	89 (70.1)	0.45
Manic/hypomanic or mixed	9 (7.1)	
None	29 (22.8)	
Polarity upon enrollment in the study, No. (%)		
Depressive	43 (33.9)	0.45
Manic/hypomanic	2 (1.6)	
Euthymic	82 (64.6)	
Patient Health Questionnaire (PHQ9) at baseline	12 (6)	
Rapid cycling, No. (%)	19 (15.0)	0.46
History of psychotic symptoms, No. (%)	47 (37.0)	0.46
History of suicide attempts, No. (%)	37 (29.1)	0.48
History of hospital admissions, No. (%)	62 (48.8)	0.48
Comorbid psychiatric diagnosis(es), No. (%)	89 (77.4)	0.37
Comorbid physical diagnosis(es), No. (%)	20 (15.7)	0.41

**Table 2:** Study cohort: description of wearable data

Variable	Mean (SD)
Daily Steps (steps)	6705.7 (3507.9)
Rest Periods (hours/day)	8.8 (2.5)
Inactivity Periods (hours/day)	7.8 (2.4)
Total Sleep Time (hours/night)	7.5 (1.3)
REM Sleep Time (hours/night)	1.6 (0.5)
Deep Sleep Time (hours/night)	1.5 (0.5)

SD: Standard Deviation

For depressive symptom labeling, we analyzed 6,985 PHQ-9 data points. Based on a PHQ-9 score ≥ 10 or higher for at least two weeks, we identified a total of 168 depressive episodes in 72 (57%) participants, for a mean (SD) of 1.47 (2.76) episodes per participant.

In keeping with our previous work (Halabi et al. 2024; Ortiz et al. 2023), the proportion of missing data was lowest for both variables measured by the wearable: (mean (SD) for activity: 2.17% (0.5%); and sleep: 2.91% (0.3). The proportion of missing data for self-rating scales was 17.8% (17.3%).

### Exploratory analysis

Upon transformation of all raw data variables into the frequency domain, we observed that both activity and sleep patterns varied at an average rate of 4-day cycles (mean (SD) f_activity_, 0.25 (0.01) Hz/day; mean (SD) f_activity_, 0.25 (0.04) Hz/day) across study participants. Computing the intrinsic mode functions (IMF) for each variable resulted in partially intersecting sub bands of activity and sleep patterns, which allowed us to monitor activity and sleep variability rates on a day-to-day scale (i.e., raw data) or every three weeks scale (i.e., IMF-3).

### Detecting the onset of depressive symptoms

**Fig. 2:**
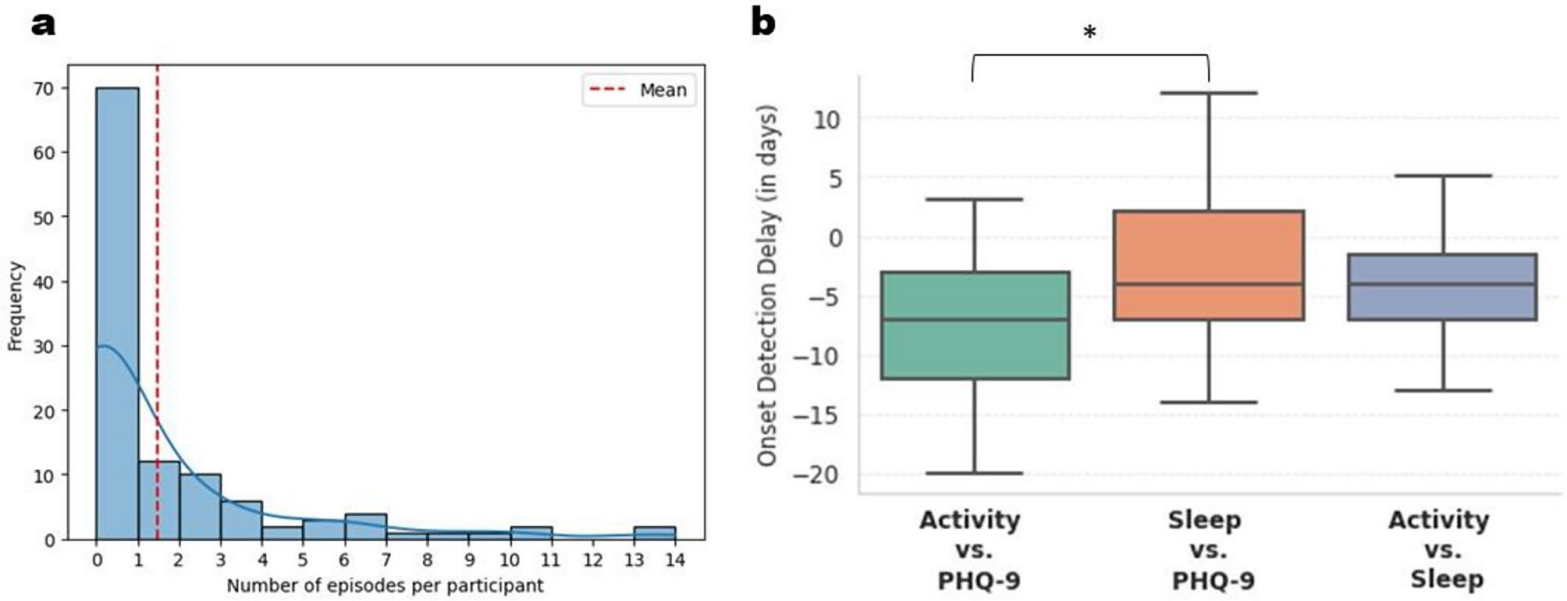
Episode distribution per participant and onset detection delay between wearable variables and PHQ-9

After extracting activity and sleep patterns at different temporal scales, we detected instances of significant instantaneous frequency (IF) fluctuations around the onset of depressive symptoms using a spectral derivative spike detection (SD^2^) algorithm. We detected the onset of depressive symptoms with a median (IQR) prediction window of -7.0 (-9.0) days using activity variables, and − 4.0 (-5.0) days using sleep variables (Fig. 2). Our definition of a prediction window is the number of days between the detection of depressive symptoms based on wearable data vs. PHQ-9 scores, such that a negative value indicates early detection, and a positive value indicates late detection (see Methods).

Using 2-week early detection sweeping (EDS), we detected the onset of depressive symptoms with highest sensitivity using raw (i.e., day-to-day) time-frequency patterns, across all activity and sleep variables. Among activity variables, the highest detection accuracy was obtained using day-to-day variability in the number of steps (mean (SD) sensitivity, 0.79 (0.27)) and minutes of inactivity (mean (SD) sensitivity, 0.79 (0.27)). Among sleep variables, the highest detection accuracy was observed using day-to-day variability of deep sleep duration (mean (SD) sensitivity, 0.80 (0.26)) and REM sleep duration (mean (SD) sensitivity, 0.80 (0.27)). Conversely, periods of euthymia were most accurately detected using the lowest temporal scale component (i.e., every three weeks) of activity (mean (SD) specificity, 0.86 (0.17)) and sleep (mean (SD) specificity, 0.96 (0.07)). Details on detection performance metrics using EDS are presented in Table 3.

**Table 3:** Depressive symptom onset detection performance metrics using 2-week early detection sweeping (EDS)

			Performance Metric (mean [SD])			
Variable	Mode	Rate	Sensitivity	Specificity	Accuracy	*P* Value
Steps	IMF-0	Daily	0.79 [0.27]	0.86 [0.17]	0.74 [0.19]	< 0.05
	IMF-1	Weekly	0.74 [0.32]	0.91 [0.07]	0.76 [0.23]	
	IMF-2	Every 2 weeks	0.67 [0.40]	0.95 [0.05]	0.78 [0.28]	
	IMF-3	Every 3 weeks	0.66 [0.41]	0.97 [0.05]	0.79 [0.29]	
Rest	IMF-0	Daily	0.78 [0.27]	0.86 [0.11]	0.74 [0.19]	< 0.05
	IMF-1	Weekly	0.73 [0.34]	0.91 [0.07]	0.76 [0.23]	
	IMF-2	Every 2 weeks	0.68 [0.39]	0.95 [0.05]	0.78 [0.29]	
	IMF-3	Every 3 weeks	0.64 [0.43]	0.96 [0.06]	0.79 [0.29]	
Inactivity	IMF-0	Daily	0.79 [0.27]	0.86 [0.10]	0.75 [0.18]	< 0.05
	IMF-1	Weekly	0.74 [0.32]	0.92 [0.07]	0.78 [0.22]	
	IMF-2	Every 2 weeks	0.68 [0.39]	0.95 [0.05]	0.78 [0.28]	
	IMF-3	Every 3 weeks	0.64 [0.43]	0.95 [0.06]	0.78 [0.30]	
Total Sleep	IMF-0	Daily	0.79 [0.27]	0.86 [0.11]	0.75 [0.20]	< 0.05
	IMF-1	Weekly	0.74 [0.32]	0.91 [0.08]	0.77 [0.23]	
	IMF-2	Every 2 weeks	0.70 [0.38]	0.95 [0.05]	0.79 [0.27]	
	IMF-3	Every 3 weeks	0.65 [0.43]	0.95 [0.07]	0.78 [0.29]	
REM Sleep	IMF-0	Daily	0.80 [0.27]	0.86 [0.11]	0.75 [0.20]	< 0.05
	IMF-1	Weekly	0.75 [0.31]	0.90 [0.09]	0.77 [0.22]	
	IMF-2	Every 2 weeks	0.69 [0.38]	0.95 [0.06]	0.79 [0.28]	
	IMF-3	Every 3 weeks	0.66 [0.42]	0.96 [0.06]	0.80 [0.28]	
Deep Sleep	IMF-0	Daily	0.80 [0.26]	0.87 [0.10]	0.75 [0.18]	< 0.05
	IMF-1	Weekly	0.75 [0.32]	0.91 [0.08]	0.79 [0.22]	
	IMF-2	Every 2 weeks	0.70 [0.38]	0.95 [0.06]	0.79 [0.27]	
	IMF-3	Every 3 weeks	0.66 [0.42]	0.95 [0.07]	0.79 [0.28]	

Legend for Table 3: Depressive symptom onset detection performance metrics using 2-week early detection sweeping (EDS). This table summarizes the mean and standard deviation (SD) of performance metrics, including sensitivity (hits), specificity (correct non-detection), accuracy, and p-values for daily, over one week and over two weeks aggregation of steps, rest, inactivity, total sleep, REM sleep, and deep sleep using various Intrinsic Mode Functions (IMFs). Significant p-values indicate statistically significant differences in performance metrics

Using symmetrical bidirectional sweeping (SBS), which covers early and late detection of depressive symptom onsets, higher detection performance was observed across all variables at all rates. As with EDS, we correctly detected the highest proportion of depressive symptoms onset using the most granular activity and sleep variable components (i.e., day-to-day time-frequency patterns). Details on detection performance metrics using SBS are presented in Table 4.

**Table 4:** Depressive symptom onset detection performance metrics using 2-week symmetrical bilateral sweeping (SBS)

			Performance Metric (mean [SD])			
Variable	Mode	Rate	Sensitivity	Specificity	Accuracy	*P* Value
Steps	IMF-0	Daily	0.77 [0.24]	0.87 [0.19]	0.76 [0.19]	< 0.05
	IMF-1	Weekly	0.73 [0.31]	0.92 [0.08]	0.78 [0.19]	
	IMF-2	Every 2 weeks	0.69 [0.42]	0.94 [0.06]	0.78 [0.28]	
	IMF-3	Every 3 weeks	0.64 [0.44]	0.97 [0.05]	0.79 [0.26]	
Rest	IMF-0	Daily	0.88 [0.22]	0.85 [0.12]	0.81 [0.17]	< 0.05
	IMF-1	Weekly	0.82 [0.31]	0.92 [0.08]	0.83 [0.19]	
	IMF-2	Every 2 weeks	0.74 [0.24]	0.94 [0.04]	0.84 [0.23]	
	IMF-3	Every 3 weeks	0.68 [0.29]	0.95 [0.04]	0.85 [0.26]	
Inactivity	IMF-0	Daily	0.81 [0.25]	0.87 [0.10]	0.77 [0.16]	< 0.05
	IMF-1	Weekly	0.76 [0.33]	0.91 [0.09]	0.79 [0.22]	
	IMF-2	Every 2 weeks	0.69 [0.38]	0.93 [0.06]	0.81 [0.24]	
	IMF-3	Every 3 weeks	0.66 [0.41]	0.94 [0.05]	0.82 [0.27]	
Total Sleep	IMF-0	Daily	0.81 [0.29]	0.87 [0.10]	0.77 [0.15]	< 0.05
	IMF-1	Weekly	0.75 [0.33]	0.91 [0.09]	0.79 [0.17]	
	IMF-2	Every 2 weeks	0.72 [0.37]	0.94 [0.04]	0.80 [0.17]	
	IMF-3	Every 3 weeks	0.67 [0.42]	0.95 [0.07]	0.81 [0.22]	
REM Sleep	IMF-0	Daily	0.82 [0.29]	0.87 [0.10]	0.76 [0.18]	< 0.05
	IMF-1	Weekly	0.78 [0.33]	0.91 [0.11]	0.79 [0.22]	
	IMF-2	Every 2 weeks	0.71 [0.39]	0.93 [0.08]	0.81 [0.24]	
	IMF-3	Every 3 weeks	0.69 [0.41]	0.96 [0.06]	0.81 [0.26]	
Deep Sleep	IMF-0	Daily	0.87 [0.23]	0.89 [0.09]	0.77 [0.14]	< 0.05
	IMF-1	Weekly	0.79 [0.35]	0.93 [0.07]	0.78 [0.18]	
	IMF-2	Every 2 weeks	0.75 [0.33]	0.94 [0.05]	0.80 [0.21]	
	IMF-3	Every 3 weeks	0.69 [0.38]	0.95 [0.04]	0.82 [0.23]	

Legend for Table 4: Depressive symptom onset detection performance metrics using 2-week Symmetrical Bilateral Sweeping (SBS). This table summarizes the mean and standard deviation (SD) of performance metrics, including sensitivity (hits), specificity (correct non-detection), accuracy, and p-values for daily, over one week and over two weeks aggregation of steps, rest, inactivity, total sleep, REM sleep, and deep sleep using various Intrinsic Mode Functions (IMFs). Significant p-values indicate statistically significant differences in performance metrics

**Fig. 3:**
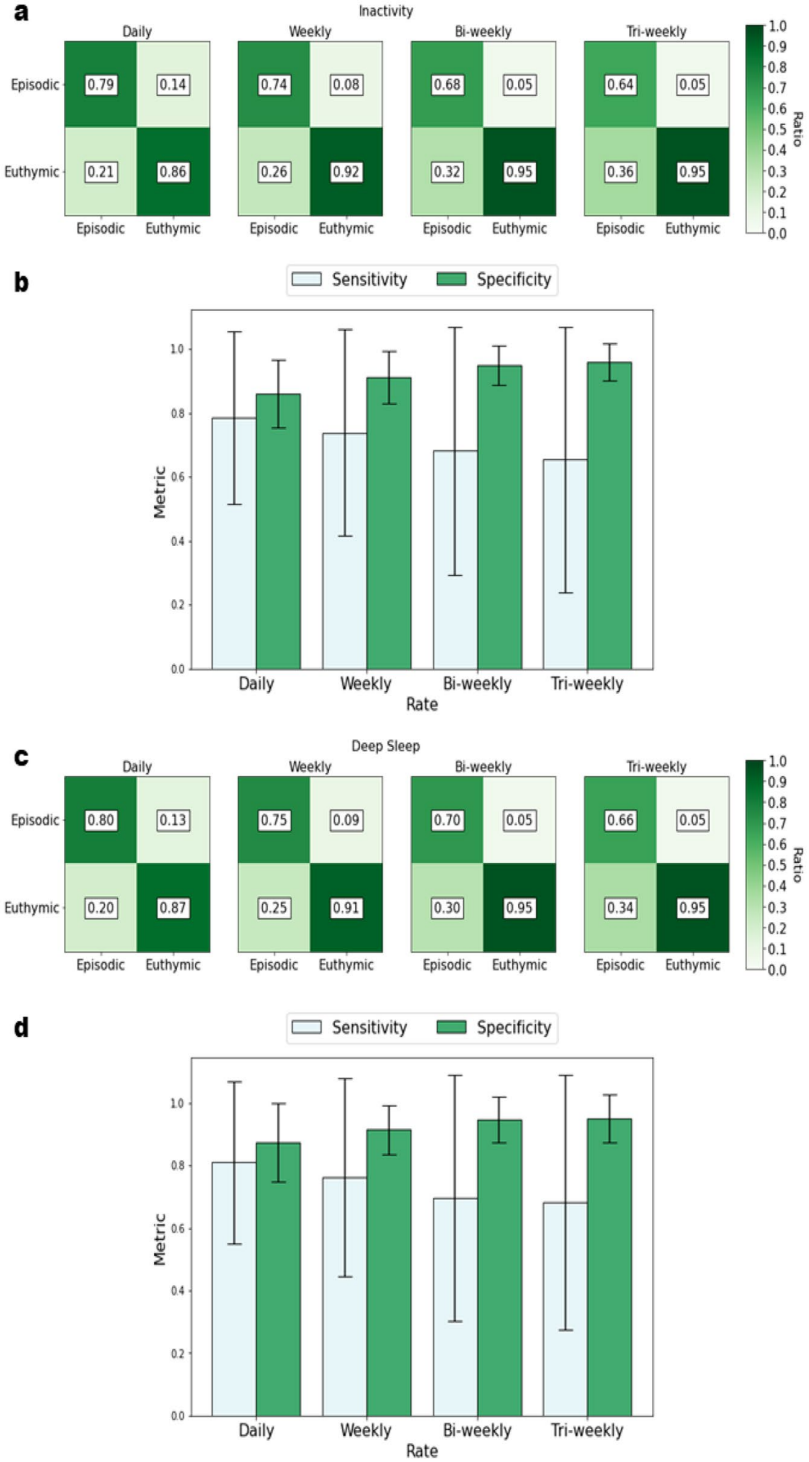
Depressive symptom detection performance metrics for **a**,** b)** inactivity and **c**,** d)** deep sleep at different rates. Legend: Early Detection Sweeping (EDS) depressive symptoms detection performance metrics for panels a & b: inactivity; and c & d: deep sleep. The confusion matrices in panels a & c illustrate the classification outcomes: the upper left quadrant shows hits (True Positive Rate, TPR), when depressive symptoms were correctly detected; the upper right quadrant shows false alarms (False Positive Rate, FPR), when depressive symptoms were detected but no episodes occurred; the lower left quadrant shows misses (False Negative Rate, FNR), when depressive symptoms were missed; and the lower right quadrant shows correct non-detection (True Negative Rate, TNR), when euthymia (i.e., absence of symptoms) was correctly identified

For activity, the day-to-day variability of the duration of rest (in minutes) provided the most accurate detection of depressive symptoms (mean (SD) sensitivity, 0.88 (0.22)); while every three weeks’ variability of daily steps provided the most accurate detection of euthymia (mean (SD) specificity, 0.97 (0.05)). For sleep, day-to-day variability in the duration of deep sleep (in minutes) provided the most accurate detection of depressive symptoms (mean (SD) sensitivity, 0.87 (0.23)); while every three weeks variability in duration of REM sleep (in minutes) provided the most accurate detection of euthymia (mean (SD) specificity, 0.96 (0.06)). Confusion matrices and statistical distributions of the variables of highest performance are presented in Fig. 3.

## Discussion

We assessed 184,179 data points in 127 patients with BD followed for over a year in an ongoing e-monitoring study that provided objective densely-sampled data to anticipate transitions to depressive symptoms. We found that changes in activity and sleep can anticipate changes in PHQ-9 by 7–9 and 4–5 days, respectively. Our study contributes to the field by developing a robust approach for analyzing densely sampled multivariate time-series data for each participant, representing an important step towards understanding variability in behavioral variables at a granular level. This is important because it allows us to better understand how symptoms change at the day-to-day level, with dynamic interactions between behavioral changes and mood regulation. An “episode” then, represents the endpoint of the long-term interplay of day-to-day variability in different systems (Wichers 2014). Our results are also consistent with the literature showing that mood and energy are two different dimensions (Merikangas et al. 2019) and confirm our previous findings (Ortiz et al. 2018) showing the importance of changes in activity in forecasting episodes of illness in BD. Our results are also congruent with findings reporting that improvement in depressive symptoms occur earlier than can be detected at weekly clinical visits (Lenderking et al. 2008).

Our results suggest that interventions targeting activity (i.e., behavioral activation) could be a critical component for the prevention of depressive episodes in BD. This is consistent with the Behavioral Activation System (BAS) hypersensitivity model (Depue et al. 1981) according to which patients with BD struggle regulating their behavior due to their proneness to BAS dysregulation, rendering them sensitive to BAS-relevant events and resulting in hypo(manic) episodes (Alloy et al. 2008). While this theory has been expanded to include a related system (Behavioral Inhibition System) and its association with depressive episodes, further studies are required to integrate these theoretical approaches in clinical practice.

Understanding the dynamic relationship between activity and mood in BD is also important for diagnostic considerations: for over 20 years, authors have suggested that changes in energy or activity are more relevant than mood changes in BD (Akiskal et al. 2003). Two large international studies(Angst et al. 2012; Machado-Vieira et al. 2017) reported that the accuracy of a diagnosis of BD was improved by emphasizing changes in energy as a core symptom of hypo(mania), in addition to changes in mood. Other studies have reported that increased activity, more than elation, is the core feature of mania (Cheniaux et al. 2014) and that clinical or actigraphy measures of activity can distinguish manic from mixed episodes.

Limitations of our study include that we could not analyze transitions to manic or hypomanic symptoms because there were not enough such symptoms in our sample; and the lack of control for sociodemographic variables. Another limitation of our study (and any similar study that collects irregularly sampled multivariate data) is the difference in sampling frequency between wearable data variables (i.e., activity, sleep) and PHQ-9 self-reports, which could introduce delays in onset detection. To address this difference in sampling frequencies, we used retrospective label-padding to backfill binary labels for the two-week period preceding the relapse. Moreover, some of the strengths that distinguished our study from published studies was the use of densely sampled data in a well-characterized cohort followed for over a year. Another strength of this study was the novel approach using time-frequency analysis to dynamically assess behavioral features at a granular level. These strengths may explain why, in contrast to all studies in BD published to date (Bos et al. 2022; Kunkels et al. 2021) our analysis showed that changes in e-monitored variables preceded changes in PHQ-9 with high sensitivity and specificity. Finally, this study was contactless (i.e., conducted remotely in its entirety), which further emphasizes the feasibility of embedding technology within a clinically informed context to improve clinical outcomes.

Future studies should explore the effect of “Just in Time Interventions” to assess how interventions dynamically targeting activity or sleep could influence mood variability (i.e., prevent mood episodes). These steps will pave the way for an era in Psychiatry that routinely employs clinically actionable predictions at the individual level.

Our study adds new knowledge to the field by developing a robust methodology to detect transitions in BD, and by showing that changes in activity levels could be used to forecast these transitions. A more dynamic representation of behavioral phenomena in mood disorders may provide a renewed emphasis on early interventions using behavioral activation in the prevention of depressive episodes in BD.

## Data Availability

The datasets generated and/or analyzed during the current study are not publicly available due to privacy considerations.

## Code availability

The underlying code for this study is not publicly available but may be made available to qualified researchers on reasonable request from the corresponding author.
